# *Mycobacterium tuberculosis* Protein Rv3841 Activates Dendritic Cells and Contributes to a T Helper 1 Immune Response

**DOI:** 10.1155/2018/3525302

**Published:** 2018-03-15

**Authors:** Seunga Choi, Han-Gyu Choi, Ki-Won Shin, Yong Woo Back, Hye-Soo Park, Jae Hwi Lee, Hwa-Jung Kim

**Affiliations:** ^1^Department of Microbiology, College of Medicine, Chungnam National University, Daejeon, Republic of Korea; ^2^Department of Medical Science, College of Medicine, Chungnam National University, Daejeon, Republic of Korea

## Abstract

The attenuated vaccine *Mycobacterium bovis* BCG (Bacille Calmette Guerin) has limited protective efficacy against TB. The development of more effective TB vaccines has focused on the mycobacterial antigens that cause strong T helper 1 (Th1) responses. Mtb protein Rv3841 (bacterioferritin B; BfrB) is known to play a crucial role in the growth of Mtb. Nonetheless, it is unclear whether Rv3841 can induce protective immunity against Mtb. Here, we studied the action of Rv3841 in maturation of dendritic cells (DCs) and its engagement in the development of T-cell immunity. We found that Rv3841 functionally activated DCs by upregulating costimulatory molecules and increased secretion of proinflammatory cytokines. Activation of DCs by Rv3841 was mediated by Toll-like receptor 4 (TLR4), followed by triggering of mitogen-activated protein kinase and nuclear factor-*κ*B signaling pathways. In addition, Rv3841-matured DCs effectively proliferated and polarized Th1 immune response of naïve CD4^+^ and CD8^+^ T-cells. Moreover, Rv3841 specifically caused the expansion of CD4^+^CD44^high^CD62L^low^ T-cells from Mtb-infected mice; besides, the T-cells activated by Rv3841-matured DCs inhibited intracellular mycobacterial growth. Our data suggest that Rv3841 induces DC maturation and protective immune responses, a finding that may provide candidate of effective TB vaccines.

## 1. Introduction

For tuberculosis (TB) to be removed from the top rank of global health problems within any practical time frame, transformative tools and projects will need to be developed [1]. The currently licensed anti-TB vaccine *Mycobacterium bovis* Bacille Calmette Guerin (BCG) confers insufficient protection from pulmonary TB in adolescents and adults [2]. Effective vaccines in latently infected individuals and adults are strongly needed.

The immunological mode of action of an effective TB vaccine involves driving the immunodominant CD4^+^ and CD8^+^ T-cell responses that can eliminate the invading bacteria. Priming and expansion of the antigen-specific T-cells after a primary *Mycobacterium tuberculosis* (Mtb) infection occur in regional lymph nodes that drain the lungs, and these responses are initiated by Mtb-infected dendritic cells (DCs) trafficking from the lungs [3, 4]. On the other hand, it has been reported that Mtb modulates the infected DCs to inhibit antigen presentation to T-cells, thus delaying recruitment of activated T-cells into the lungs from lymph nodes [5]. Therefore, effective DC activation and migration are necessary to eliminate Mtb via an adaptive immune response.

DCs are the most potent antigen-presenting cells in terms of activation of naïve T-cells and play a critical role in the initiation of both primary and secondary immune responses to pathogens [6, 7]. DCs express diverse cell surface markers, and phenotypic analysis broadly classifies DCs into immature and mature stages [8]. Mature DCs show high expression of costimulatory molecules, such as CD40, CD80, and CD86, as well as MHC class II antigens [9]. This maturation can be caused by stimuli, such as tumor necrosis factor *α* (TNF-*α*), interleukin-1*β* (IL-1*β*), and components of infectious agents. Many stimuli, related maturation, induce phosphorylation of mitogen-activated protein kinases (MAPKs), such as p38 MAPK, c-Jun N-terminal kinases (JNKs), and extracellular signal-regulated kinases (ERKs). Then, the MAPK signaling pathways have obvious functions in the DC maturation [9]. Recent studies suggest that MAPK signaling pathways differentially regulate appearances of phenotypic maturation, cytokine production, and functional differentiation of DCs [10–12]. Thus, distinct maturation of DCs may be induced by modulating the balance of phosphorylation of MAPKs.

Immature DCs can capture and internalize mycobacterial antigens that engage surface-expressed receptors, such as Toll-like receptors (TLRs) [13]. The innate immune response prompt by involvement of TLRs implicates recruitment of cytoplasmic adaptor proteins and signaling molecules, resulting in phagosome maturation and the production of proinflammatory cytokines [13, 14]. Many studies have shown that mycobacterial antigens participate in innate recognition and responses through TLR signaling [15–17]. TLR receptors activate signal transduction cascades that sequentially activate the adaptor protein myeloid differentiation factor 88 (MyD88) and tumor necrosis factor receptor 6, eventually promoting the translocation of NF-*κ*B and activation of MAPKs [18].

Critical players in antimycobacterial immune responses are T lymphocytes and antigen-presenting cells including macrophages and DCs. The mycobacterial antigen-activated DCs may preferentially drive CD4^+^ T helper cells to polarize into T helper 1 (Th1) or Th2 cell types, thereby controlling the development of type 1 or type 2 immune responses [19, 20]. Immunological control of Mtb infection is based on a type 1 T-cell response [21]. IL-12 is induced after phagocytosis of Mtb by macrophages and DCs [22, 23]; this process drives the development of a Th1 response with production of IFN-*γ*. Th1 immune responses are relevant to containment and control of Mtb replication and involve IFN-*γ*-producing or polyfunctional (IL-2-, IFN-*γ*-, and TNF-*α*-producing) CD4^+^ and CD8^+^ T lymphocytes [24]. Although many mycobacterial proteins that cause DCs to secrete proinflammatory cytokines have been identified [17], DC-activating antigens with a proven protective effect against Mtb are rarely reported. We hypothesized that the proteins that induce maturation of DCs and a Th1 immune response can be potential candidate for use in vaccines against Mtb.

Protein Rv3841 (also known as BfrB, Mtb ferritin B) is involved in iron storage. Ferritins are known as important participant in iron storage and detoxification processes linked to Mtb's growth and pathogenicity [25–27]. Therefore, we theorized that Rv3841 is a good target for the development antitubercular drugs and vaccine candidates. In this study, we report that recognition of recombinant Rv3841 by DCs causes these cells to mature and to promote a Th1 immune response. Our results suggest that by acting on antigen-presenting cells, such as DCs, recombinant Rv3841 proteins may regulate immune responses to Mtb and the relevant host defense mechanism.

## 2. Materials and Methods

### 2.1. Animals

5-6 weeks of age, female C57BL/6 TLR2 knockout (KO) mice, TLR4 KO mice, C57BL/6 OT-I mice, and OT-II T TCR transgenic mice were purchased from the Jackson Laboratory (Bar Harbor, ME, USA). All animals were maintained under specific pathogen-free (SPF) barrier conditions at Preclinical Research Center (PCRC) of Chungnam National University Hospital, Daejeon, Korea. All mice used are in accordance with the guide for care and used of the Korean Food and Drug Administration (KFDA). All animal studies used were licensed by the Ethics Committee and Institutional Animal Care and Use Committee (Permit number 2014-0197-3) of the Laboratory Animal Research Center at IACUC (CNU-00284) of Chungnam National University (Daejeon, Korea).

### 2.2. Generation of Mouse Bone Marrow-Derived Dendritic Cells and Macrophages

Bone marrow-derived dendritic cells (BMDCs) and bone marrow-derived macrophages (BMDMs) were differentiated in vitro from isolated bone marrow cells from uninfected 5-6-week-old C57BL/6 mice. The cells were generated and cultured as recently described [28]. Briefly, using BMDC differentiation method, bone marrow cells collected from mouse femurs and tibias were incubated for 7 d in RPMI media supplemented with 10% fetal bovine serum (FBS), penicillin/streptomycin (100 unit/mL), nonessential amino acids (0.1 mM), *β*-mercaptoethanol (50 *μ*M), sodium pyruvate (1 mM), GM-CSF (20 ng/mL), and IL-4 (10 ng/mL). For the differentiation of BMDMs, bone marrow cells were cultured in DMEM media containing 10% FBS and 20 ng/mL of M-CSF for 6 d.

### 2.3. Purification of Rv3841 Protein and Confirmation of LPS Decontamination

Rv3841 (BfrB) gene was amplified by PCR using the Mtb H37Rv ATCC27294 genomic DNA as a template and the following primers: Rv3841 forward, 5′-CATATGACAGAATACGAAGGGCCTAAG-3′, and reverse, 5′-AAGCTTGAGGCGGCCCCCGGCAGCGTG-3′. The PCR product of Rv3841 was cloned with the pET22b (+) (Novagen, Madison, WI, USA) with His tagged at the C-terminus. *E. coli* BL21 bacteria carrying Rv3841 expressed plasmid was induced with IPTG (isopropyl-*β*-D-thiogalactopyranoside). His-tagged recombinant Rv3841 protein was purified with Ni-NTA columns (Qiagen, Valencia, CA). The purification protocol was performed as previously described [29]. To confirm the contaminating endotoxins or LPS in protein purification, digestion with proteinase K (Sigma), pretreatment with polymyxin B (PmB) (Sigma), and heat denaturation were performed.

### 2.4. Flow Cytometry Analysis

To investigate the cytotoxic effect of Rv3841 on the DCs, DCs (1 × 10^6^ cells/mL) were incubated with 10 *μ*g/mL Rv3841 for 24 h. After 24 h of treatment, the cells were stained with FITC-conjugated annexin V and PI from R&D Systems (Minneapolis, MN, USA). Cell toxicity was detected according to the manufacturer's instructions. Then, samples were detected on the FACSCanto II with FACSDiva and analyzed using the FlowJo software (Tree Star, Ashland, OR, USA). To investigate the surface molecules, after 24 h of Rv3841 protein treatment, the cells were stained with PE-conjugated anti-CD80, anti-CD86, anti-H-2Kb (MHC class I), and anti-I-Ab (MHC class II) with FITC-conjugated anti-CD11c antibodies from eBioscience (San Diego, CA, USA) for 30 min at 4°C. The fluorescence was measured by flow cytometry.

### 2.5. Immunoblotting Analysis

Immunoblotting (IB) was performed as described previously [29]. Briefly, cells seeded at 10^6^ cells/mL in 6-well plates were treated with or without LPS or Rv3841 protein. At 24 h incubation, the cells were harvested and lysed in cell lysis buffer (50 mM Tris HCl, pH 8.0; 137 mM NaCl; 1 mM EDTA; 1% (vol/vol) Triton X-100; 10% (vol/vol) glycerol; 1 mM PMSF; 1 *μ*g/mL each of aprotinin, leupeptin, and pepstatin; 1 mM Na3VO4; and 1 mM NaF). The cell lysates from each sample were subjected to SDS-PAGE followed by the transfer of proteins to PVDF membranes. ECL reagents (Millipore) were applied for immune blot analysis.

### 2.6. Treatment of DCs with Pharmacological Inhibitors of Signaling Pathways

All the pharmacological inhibitors were purchased from Calbiochem (San Diego, CA, USA). Dimethyl sulfoxide (Sigma) was added to cultures at 0.1% (vol/vol) as a solvent control. Inhibitors were used at the following concentrations: U0126 (10 *μ*M), SB203580 (20 *μ*M), SP600125 (10 *μ*M), and Bay11-7082 (20 *μ*M). A tested concentration was used after determining the viability of DCs in titration experiments using an MTT assay. In experiments with inhibitors, the cells were treated with a given inhibitor for 1 h before treatments with proteins.

### 2.7. In Vitro T-Cell Proliferation Assay

For T-cell proliferation assay, OVA-specific CD4^+^ and CD8^+^ T-cells were isolated using a MACS column (Miltenyi Biotec, Bergisch Gladbach, Germany) from splenocytes of OT-I and OT-II transgenic mice. Then, these T-cells were stained with 1 *μ*M CFSE (Invitrogen). T-cell proliferation assay was performed as recently described [28]. Briefly, DCs were treated with the OVA peptide from Peptron (Daejeon, Korea) and 10 *μ*g/mL of Rv3841 for 24 h. After that, Rv3841-activated DCs were cocultured with CFSE-stained T-cells at DC : T-cell ratios of 1 : 10. After 3 d of coculture, each T-cell batch was stained with anti-CD4^+^ mAb or anti-CD8^+^ mAb from eBioscience analyzed by flow cytometry. The supernatants were harvested and measured by ELISAs from eBioscience.

### 2.8. Bacteria, Mtb Infection in Mice, and Cell Preparation

Virulent Mtb strain H37Rv ATCC 27294 and the avirulent strain H37Ra ATCC 25177 were purchased from American Type Culture Collection (ATCC, Manassas, VA). All mycobacteria were provided from the International Tuberculosis Research Center (ITRC, Changwon, Gyeongsangnam-do, South Korea). These strains were cultured in Middlebrook 7H9 broth (Difco Laboratories, Detroit, MI) supplemented with 0.02% glycerol and 10% (vol/vol) oleic acid-albumin-dextrose-catalase (OADC, Becton Dickinson, Sparks, MD) for 25–28 days at 37°C. Age- and sex-matched C57BL/6 mice were infected with Mtb H37Ra, and the mycobacteria preparation protocol was performed as previously described [29]. Briefly, 6-week-old mice per group were intravenous initial infectious dose with 10^7^ CFU Mtb H37Ra. The infected mice were euthanized at 6 weeks after infection to analyze immune responses. CD4^+^ T-cells were isolated from the spleens of H37Ra-infected mice using a MACS column.

### 2.9. Analysis of the Activation of Effector/Memory T-Cells

For memory response analysis, C57BL/6 mice at 6 weeks of age were infected with Mtb H37Ra as described above. DCs (2 × 10^5^ cells/well) isolated from WT C57BL/6 mice were treated with Rv3841 for 24 h followed by extensive washing and were cocultured with 2 × 10^6^ splenocytes from Mtb-infected mice at DC : T-cell ratios of 1 : 10. On 4 days of coculture, the cells were stained with PerCP-Cy5.5-conjugated anti-CD4^+^ mAb, FITC-conjugated anti-CD62L mAb, and PE-conjugated anti-CD44 mAb from eBioscience and analyzed by flow cytometry.

### 2.10. Analysis of Cytokines

Cytokines were quantified in culture supernatants using a sandwich enzyme-linked immunosorbent assay (ELISA) as described previously [28].

### 2.11. Intracellular Staining Assays

The cells were harvested and stained for cell surface antigens CD4. After washing, cells were fixed and permeabilized, using Cytofix/Cytoperm kit (BD Biosciences), and then stained for T-bet, GATA-3, and Foxp3 fluorescein-conjugated antibodies from eBiosciences. The cells were analyzed by means of a flow cytometer.

### 2.12. Bacterial Counts

Intracellular Mtb growth assays were performed as described previously [28]. Briefly, BMDMs were seeded at 2 × 10^5^ cells/well in 24-well plates and further were infected with Mtb at MOI = 1 for 4 h. The infected BMDMs were added with 200 *μ*g/mL amikacin for 2 h to remove extracellular mycobacteria after infection. After that, a prepared T-cell mixture was added to each well and incubated for 3 d. The T-cell mixture was CD4^+^ T-cells cocultured for 3 d with antigen-activated DCs (DC : T-cell ratio = 1 : 10). The number of internalized mycobacteria within the BMDM was measured by lysing the infected cells. The bacterial counts were inspected by serial dilution on 7H10 agar (Difco Laboratories) supplemented with 0.05% glycerol and 10% OADC at 37°C. At the end of the 3 weeks, colony-forming units (CFUs) were counted from the number of colonies in plate.

### 2.13. Statistical Analysis

All experiments were performed at least three times. Statistical significance between samples was assessed with one-way ANOVA followed by Tukey's multiple comparison test using statistical software (GraphPad Prism Software, version 5.01; GraphPad Software, San Diego, CA, USA). The data represent the mean ± SEM. ^∗^ *p* < 0.05, ^∗∗^ *p* < 0.01, and ^∗∗∗^ *p* < 0.001 were considered statistically significant.

## 3. Results

### 3.1. Purification and Cytotoxicity of the Recombinant Rv3841 Protein

Rv3841 was expressed as a His-tagged protein in *E. coli* and purified by Ni-NTA affinity chromatography. The SDS-PAGE and Western blot analysis of the purified recombinant Rv3841 are shown in Figure S1A. The purified protein appeared as a major band of approximately 25 kDa, which is the expected size, according to the calculated molecular weight corresponding to the full-length amino acid sequence. To remove any contaminating endotoxins from the protein preparations, the purified Rv3841 was passed through a polymyxin B agarose column for all the experiments. The purity of Rv3841 was quantified by Quantity One software (Bio-Rad, Hercules, CA, USA) and calculated by dividing the intensity per square millimeter of the Rv3841-specific band by that of all the protein bands in the preparation lane. Rv3841 had 95% purity when 20 *μ*g of the protein preparation was stained by Coomassie staining. To determine whether Rv3841 cytotoxicity was affected DC maturation, we tested the Rv3841 protein-induced cytotoxicity in DCs by treating cells with 1, 5, and 10 *μ*g/mL Rv3841 for 24 h, then staining with annexin V, and propidium iodide to assess cell viability. Rv3841 was not cytotoxic at a concentration of 10 *μ*g/mL, indicating that a concentration below 10 *μ*g/mL would not skew the subsequent experiments (Figure S1B). Consequently, endotoxin content was measured by an LAL assay and was below 15 pg/mL (<0.1 UE/mL) in Rv3841 preparations (Figure S2A).

### 3.2. Rv3841 Induces DC Maturation

**Figure 1:**
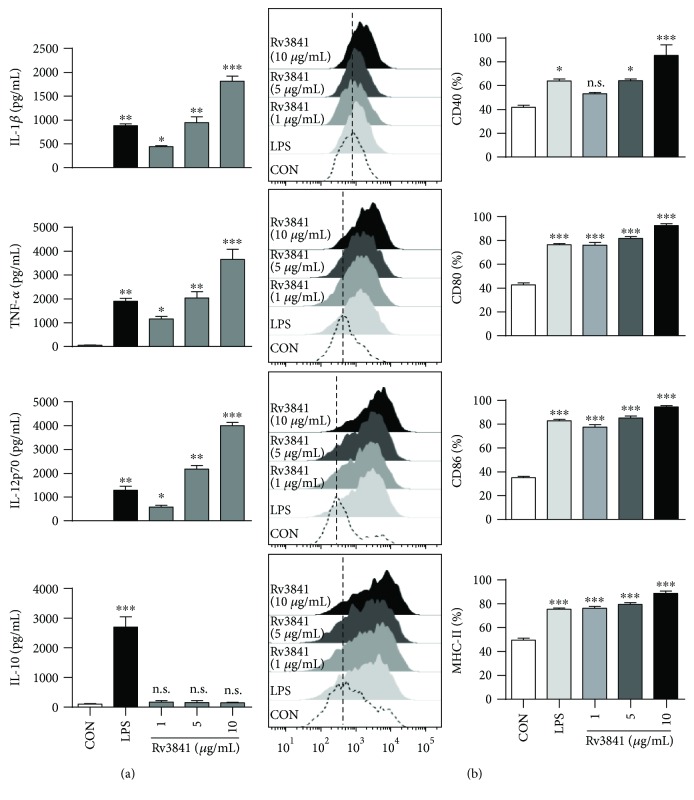
Rv3841 induces DC maturation. (a) Rv3841 induced functional activation of DCs in a dose-dependent manner. Immature DCs (10^6^ cells/mL) were cultured in the presence of 1, 5, or 10 *μ*g/mL Rv3841 or 100 ng/mL LPS for 24 h. The quantities of TNF-*α*, IL-1*β*, IL-10, and IL-12p70 in the culture supernatant were determined by ELISAs. All the data were expressed as mean ± SD (*n* = 3). The levels of significance (^∗^ *p* < 0.05, ^∗∗^ *p* < 0.01, or ^∗∗∗^ *p* < 0.001 determined by one-way ANOVA) of the differences between the treatment data and the control data are indicated; treatments that were not significantly different are indicated by “*n*.*s*.” (b) Rv3841 induced phenotypic and functional activation of DCs in a dose-dependent manner, and the cells were analyzed for the expression of surface markers by flow cytometry. The cells were gated on CD11c^+^. The DCs were stained with an anti-CD80, anti-CD86, anti-MHC class I, or anti-MHC class II antibody. The percentage of positive cells is shown in each panel. The bar graphs depict data as mean ± SD (*n* = 3). The levels of significance (^∗^ *p* < 0.05 or ^∗∗∗^ *p* < 0.001, determined by one-way ANOVA) of the differences between the treatment data and the control data are indicated. Treatments without a significant effect are indicated by “n.s.”

We first tested whether the recombinant Rv3841 could promote maturation of DCs. Immature bone marrow-derived dendritic cells were prepared by culturing for 7 days with granulocyte macrophage colony-stimulating factor (GM-CSF) and IL-4 under standard conditions and then maturated by 24 h incubation in the presence of 1, 5, or 10 *μ*g/mL Rv3841 or LPS (as a positive control). Because maturation of DCs and T-cell polarization are influenced by a variety of cytokines secreted by DCs, the levels of secretion of immunomodulatory cytokines after stimulation of immature DCs with Rv3841 were determined. We found that Rv3841 indeed caused DCs to secrete the immunomodulatory cytokines, including TNF-*α*, IL-1*β*, and IL-12p70 in a dose-dependent manner (Figure 1(a)). Nevertheless, the level of IL-10 production in Rv3841-treated DCs did not increase. We next analyzed the phenotypic alteration of DCs by analyzing the expression of various cell surface markers of DC maturation. Significant upregulation of some surface markers, including CD80, CD86, MHC class I, and MHC class II, was induced by the stimulation of DCs with Rv3841 in a concentration-dependent manner (Figure 1(b)). These results suggested that Rv3841-induced DC maturation is a potent activator of the Th1 immune response.

Several lines of evidence indicated that Rv3841-induced DC maturation was not due to contaminating endotoxins or lipopolysaccharide (LPS). For all the experiments, we used purified Rv3841 protein preparations that were passed through a polymyxin B agarose column. Furthermore, we assessed endotoxin or LPS contamination by heat denaturation and treatment with proteinase K or polymyxin B. Heat denaturation and proteinase K pretreatment abrogated the ability of the Rv3841 protein to induce DC maturation. Polymyxin B treatment did not affect the functionality of the Rv3841 protein but changed the functionality of LPS (Figure S2B). These results indicated that the maturation of DCs was induced by the intact Rv3841 protein and not by contaminating endotoxins.

### 3.3. Activation of the MAPK and NF-*κ*B Pathways Is Necessary for Rv3841-Mediated Maturation of DCs

**Figure 2:**
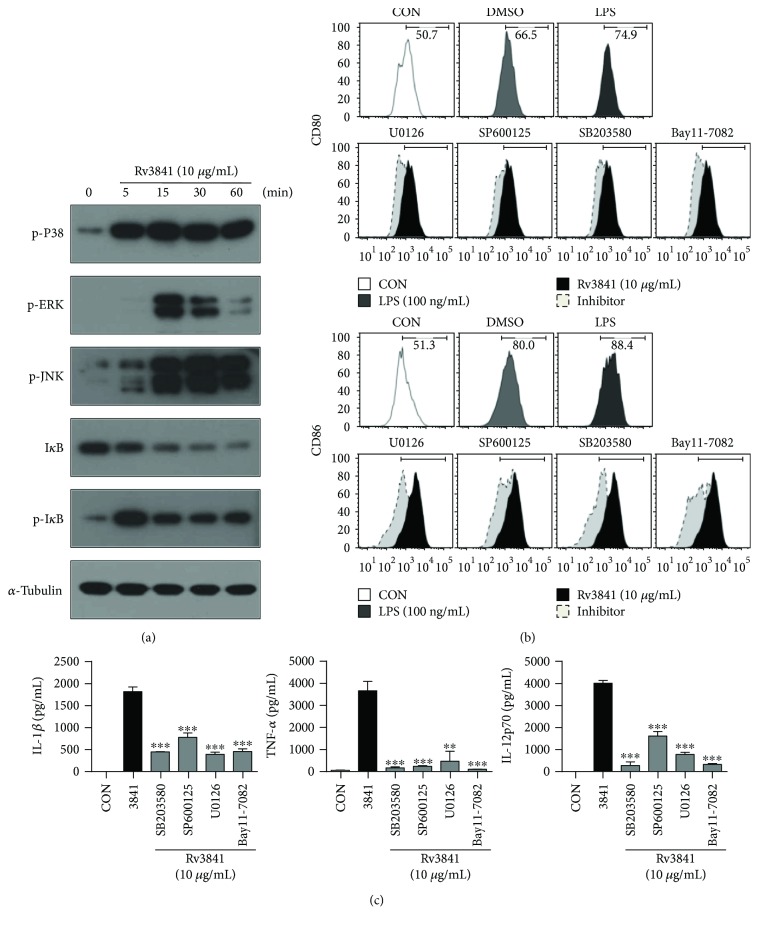
DC maturation triggered by Rv3841 involves activation of MAPKs and NF-*κ*B. (a) Protein production over time by DCs treated with 10 *μ*g/mL Rv3841. Cell lysates were subjected to SDS-PAGE, and immunoblotting analysis was carried out by means of antibodies specific to phospho-p38 (p-p38), p-ERK1/2, p-JNK, p-I*κ*B-*α*, and I*κ*B-*α*. *α*-Tubulin served as the loading control for the cytosolic proteins. Representative blots from five independent experiments are shown. (b, c) DCs were treated with pharmacological inhibitors of p38 (SB203580, 20 *μ*M), ERK1/2 (U0126, 10 *μ*M), JNK (SP600125, 20 *μ*M), or NF-*κ*B (Bay 11-7082, 20 *μ*M) or with DMSO (vehicle control) for 1 h prior to treatment with 10 *μ*g/mL Rv3841 for 24 h. (b) The expression of costimulatory molecules was determined by flow cytometry. (c) The concentrations of TNF-*α*, IL-1*β*, and IL-12p70 in the culture media were determined by ELISAs. Mean values ± SD (*n* = 3) are shown; ^∗∗^ *p* < 0.01 or ^∗∗∗^ *p* < 0.001: a significant difference from Rv3841-treated DCs, as determined by unpaired Student's *t*-test.

It has been reported that the mycobacterial antigen-mediated DC maturation is driven by activation of NF-*κ*B and MAPK pathways [10, 12]. We examined the activation of NF-*κ*B and MAPKs in response to Rv3841 treatment. Phosphorylation of MAPKs and phosphorylation and degradation of I*κ*B-*α* in DCs stimulated with Rv3841 were analyzed at the indicated time points (Figure 2). As shown in Figure 2(a), Rv3841 triggered the activation of JNK, ERK1/2, and p38. In addition, Rv3841 induced the phosphorylation and degradation of I*κ*B-*α*. The functions of these kinases in the DC maturation were corroborated by means of specific pharmacological inhibitors. Rv3841-induced expression of surface molecules (CD80 and CD86; Figure 2(b)) and proinflammatory-cytokine production (TNF-*α*, IL-1*β*, and IL-12p70; Figure 2(c)) were significantly inhibited in the cells pretreated with a p38 inhibitor (SB203580), ERK1/2 inhibitor (U0126), JNK inhibitor (SP600125), or NF-*κ*B inhibitor (Bay 11-0782) for 60 min. These findings clearly indicate that activation of MAPK and KF-*κ*B is required for the production of proinflammatory cytokines and expression of costimulatory molecules during Rv3841-mediated DC maturation.

### 3.4. Rv3841-Induced DC Maturation Is Mediated by TLR4

**Figure 3:**
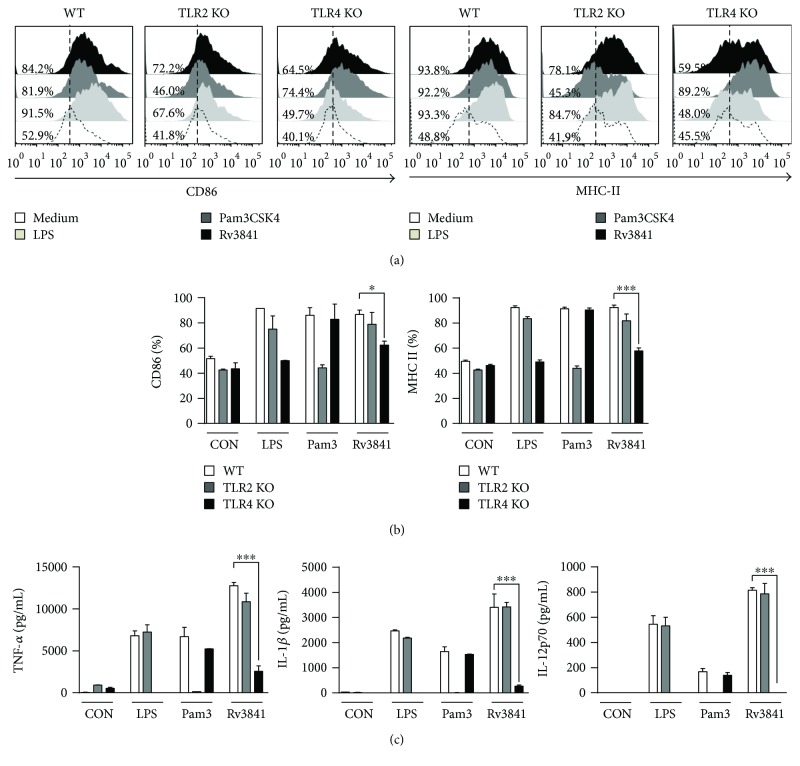
Rv3841 induces DC activation via TLR4. (a, b) Bar graphs showing the level of CD86 or MHC class II expression on Rv3841-treated CD11c^+^-gated DCs derived from WT, TLR2^−/−^, or TLR4^−/−^ mice. DCs derived from WT, TLR2^−/−^, or TLR4^−/−^ mice were treated with Rv3841 (10 *μ*g/mL) for 24 h. The percentage of positive cells is shown in each panel. The bar graphs present the mean percentage ± SEM for each surface molecule on CD11c^+^ cells across three independent experiments. ^∗∗∗^ *p* < 0.001, as determined by one-way ANOVA. (c) DCs derived from WT, TLR2^−/−^, or TLR4^−/−^ mice were treated with Rv3841 (10 *μ*g/mL) or LPS (100 ng/mL) for 24 h. The TNF-*α*, IL-1*β*, or IL-12p70 production of Rv3841- or LPS-treated DCs derived from WT, TLR2^−/−^, or TLR4^−/−^ mice was quantified by ELISAs. All the data were expressed as mean ± SD (*n* = 3); ^∗^ *p* < 0.05 or ^∗∗∗^ *p* < 0.001: a significant difference of Rv3841-treated TLR4^−/−^ DC groups from Rv3841-treated WT DC control groups and other controls, as determined by one-way ANOVA.

Mtb and its components encounter innate immunity, which operates through a variety of germline-encoded pattern recognition receptors including Toll-like receptors (TLRs) for recognition of various molecular patterns of mycobacteria [13, 17]. Therefore, we tested whether Rv3841 could be recognized by and act through TLRs in DCs. To identify TLRs interacting with Rv3841, DCs isolated from WT, TLR2^−/−^, or TLR4^−/−^ mice were stimulated with the Rv3841 protein. The expression of CD86 and MHC class II molecules (Figures 3(a) and 3(b)) and production of proinflammatory cytokines (Figure 3(c)) in TLR4^−/−^ DCs stimulated with Rv3841 were significantly weaker when compared to WT or TLR2^−/−^ DCs stimulated with Rv3841. These results clearly indicated that Rv3841 induced DC maturation in a TLR4-dependent manner.

### 3.5. Rv3841-Stimulated DCs Promote Naïve T-Cell Proliferation and Th1 Polarization

**Figure 4:**
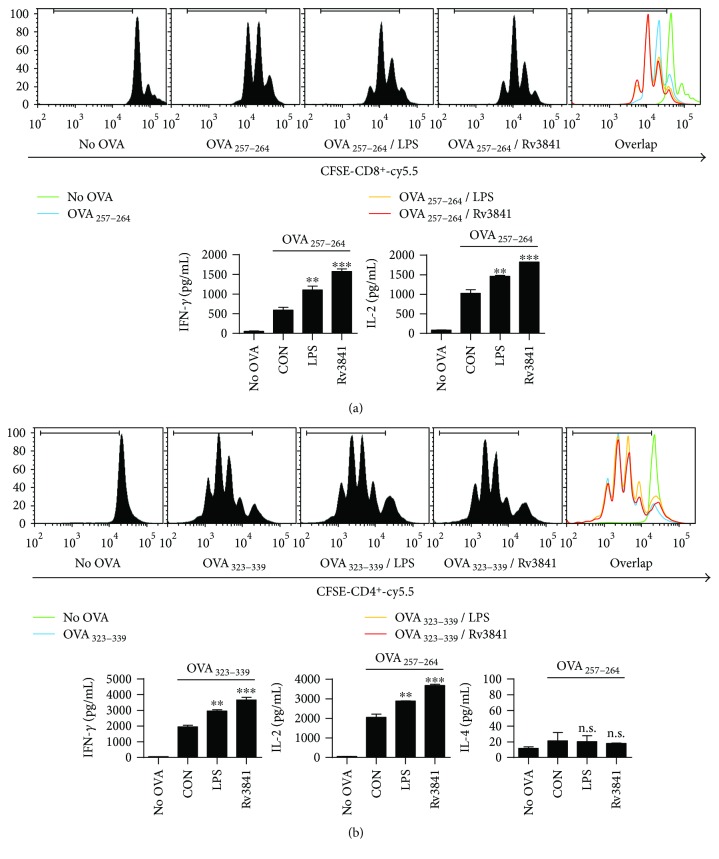
Rv3841-treated DCs stimulate T-cells to produce Th1 cytokines. (a, b) Transgenic OVA-specific CD4^+^ T-cells were isolated using MACS from OT-I and OT-II mouse splenocytes, were stained with CFSE, and were cocultured for 96 h with DCs that had been treated with Rv3841 (10 *μ*g/mL) or LPS (100 ng/mL) and then pulsed with OVA_257–264_ (1 *μ*g/mL) or OVA_323–339_ (1 *μ*g/mL) to produce OVA-specific CD4^+^ T-cells. (a) The proliferation of OT-I T-cells was then assessed by flow cytometry. T-cells alone or T-cells cocultured with untreated DCs served as the controls. Representative histograms from three independent experiments are shown. Culture supernatants were harvested after 96 h, and the IFN-*γ*, IL-2, and IL-4 concentrations were determined by ELISAs. The mean values ± SD (*n* = 3) are shown; ^∗∗^ *p* < 0.01 or ^∗∗∗^ *p* < 0.001: a significant difference of treatment groups from the appropriate controls (T-cells + OVA_257–264_-pulsed DCs or T-cells + OVA_323–339_-pulsed DCs), as determined by one-way ANOVA. Treatments without a significant effect are indicated by “n.s.”

DCs are currently considered the most efficient inducers of activation of naïve T-cells [30]. It is now clear that cessation of bacterial growth correlates with the arrival of IFN-*γ*-producing Th1-polarized CD4^+^ T-cells [31, 32] in the lungs and that a loss of CD4^+^ T-cells increases the likelihood of succumbing to tuberculosis [30, 33]. To precisely characterize the effect of T-cells interacting with Rv3841-activated DCs, we examined a T-cell proliferation assay using OT-I mouse TCR transgenic CD8^+^ T-cells and OT-II mouse TCR transgenic CD4^+^ T-cells. Transgenic CFSE-labeled OVA-specific CD4^+^ and CD8^+^ T-cells cocultured with Rv3841-treated DCs pulsed with peptide OVA_257–264_ or OVA_323–339_ proliferated to a significantly greater extent than did the same T-cells cocultured with DCs without Rv3841 treatment but pulsed with OVA_257–264_ or OVA_323–339_ (Figure 4). In addition, the secretion of IFN-*γ* and IL-2—as a consequence of priming of naïve CD4^+^ T and CD8^+^ T-cells by Rv3841-treated DCs—also significantly increased, whereas a comparable level of IL-4 secretion was not detected regardless of Rv3841 stimulation (Figure 4). FACS analysis also revealed that naïve CD4^+^ T-cells in the presence of Rv3841-treated DCs showed an increased percentage of IFN-*γ*-positive cells as compared to incubation with untreated DCs, whereas no change was observed in IL-4-positive cells (Figure S3).

**Figure 5:**
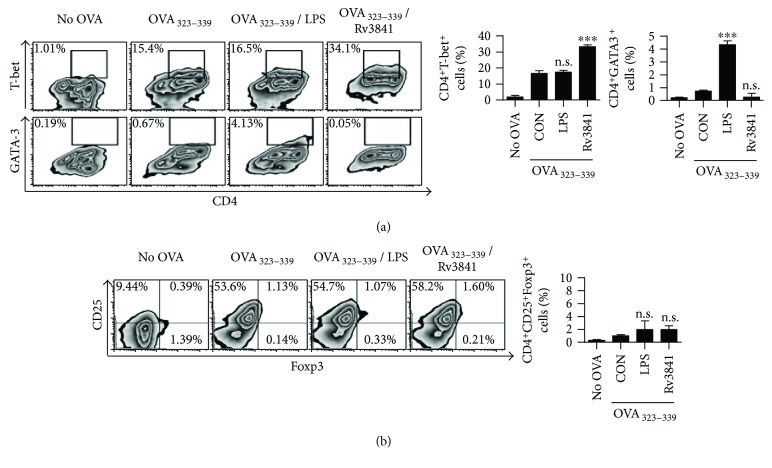
Rv3841-treated DCs stimulate T-cells to differentiate into the Th1 but not Th2 or Treg lineage. Transgenic OVA-specific CD4^+^ T-cells were isolated using MACS from OT-II mouse splenocytes and were cocultured for 72 h with DCs that were pretreated with Rv3841 (10 *μ*g/mL) or LPS (100 ng/mL) and then pulsed with OVA_323–339_ (1 *μ*g/mL) to produce OVA-specific CD4^+^ T-cells. T-cells alone or T-cells cocultured with untreated DCs served as controls. (a, b) The expression of transcription factors was then assessed using intracellular FACS analysis. The mean values ± SD (*n* = 3) are shown; ^∗∗∗^ *p* < 0.001: a significant difference of treatment groups from the appropriate controls (T-cells+OVA_323–339_-pulsed DCs), as determined by one-way ANOVA. Treatments without a significant effect are indicated by “n.s.”

Furthermore, we found that Rv3841-stimulated DCs elevated the expression of T-bet, which is a Th1-specific transcription factor, whereas the expression of GATA3, which is essential for Th2 development, was not observed (Figure 5(a)). We next analyzed the differentiation of CD4^+^CD25^+^Foxp3^+^ regulatory T-cells (Treg cells) under the influence of Rv3841-stimulated DCs. Rv3841-treated DCs did not affect the regulatory T-cell population (Figure 5(b)). These findings suggested that Rv3841-matured DC promoted proliferation of naïve T-cells and pushed them toward a Th1 phenotype.

### 3.6. Rv3841-Stimulated DCs Induce Development of Effector/Memory T-Cells

**Figure 6:**
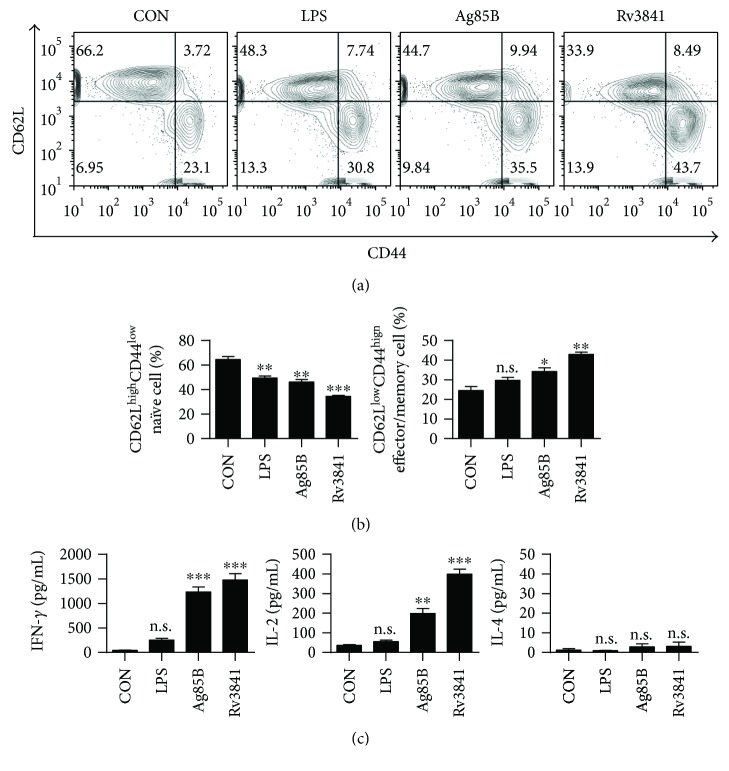
Rv3841-treated DCs induce expansion of the effector/memory T-cell population. DCs from WT mice were treated with Rv3841 (10 *μ*g/mL) or LPS (100 ng/mL) and then cocultured for 3 days with T-cells from Mtb-infected mice at the DC to T-cell ratio of 1 : 10. Splenocytes were stained with anti-CD4, anti-CD62L, and anti-CD44 monoclonal antibodies. (a, b) A histogram is shown for gating of the labeled T-cells. Bar graphs show CD62L^low^CD44^high^ T-cells or CD62L^high^CD44^low^ T-cell populations among the spleen cells. (c) Culture supernatants were harvested after 96 h, and IFN-*γ*, IL-2, and IL-4 concentrations were measured by ELISAs. The mean values ± SD (*n* = 3) are shown; ^∗^ *p* < 0.05, ^∗∗^ *p* < 0.01 or ^∗∗∗^ *p* < 0.001: a significant difference of treatment groups from the appropriate controls, as determined by one-way ANOVA test. Treatments without a significant effect are indicated by “n.s.”

To validate the properties of Rv3841 as a T-cell antigen, we determined whether Rv3841-stimulated DCs can induce expansion of the effector/memory CD4^+^ T-cell populations in Mtb-infected mice. We analyzed the surface expression of CD62L and CD44 on CD4^+^ T-cells using flow cytometry. The CD4^+^ T-cells from the spleen of Mtb-infected mice at 6 weeks postinfection were cocultured with Rv3841-treated DCs. Ag85B served as a positive control antigen because it is expressed primarily during the early stages of Mtb infection and is recognized by T-cells [34]. As shown in Figures 6(a) and 6(b), the Rv3841-treated DCs specifically induced the expansion of effector/memory T-cells by significantly downregulating CD62L and upregulating CD44 in CD4^+^ T-cells from the spleen of Mtb-infected mice when compared with untreated DCs or Ag85B- or LPS-treated DCs. In addition, the production of IFN-*γ* by the T-cells cocultured with Rv3841-treated DCs was significantly higher in comparison with T-cells cocultured with untreated DCs or LPS-stimulated DCs. IL-2 production by T-cells cocultured with Rv3841-treated DCs was significantly higher than that by T-cells cocultured with Ag85B- or LPS-treated DCs (Figure 6(c)). In contrast, IL-4 production by T-cells cocultured with Rv3841 or Ag85B-stimulated DCs remained at a baseline level. Recent studies have revealed that adaptive immune responses to *Mycobacterium tuberculosis* are delayed, including a delayed migration of dendritic cells from the lungs to the local lymph node and subsequent interaction with regulatory T-cells [3, 35–37]. Therefore, we determined whether Rv3841-stimulated DCs can induce effector/memory CD4^+^ T-cells from the lymph nodes of Mtb-infected mice (Figure S4). Interestingly, our results showed that Rv3841-stimulated DCs specifically expanded a population of CD62L^low^CD44^high^CD4^+^ effector/memory T-cells from lymph nodes of Mtb-infected mice like splenic T-cells. Although the onset of adaptive immune responses in Mtb infection is considerably delayed, our results showed that Rv3841 can act as a specific recall antigen during the course of Mtb infection. These data suggest that Rv3841-stimulated DCs induced the development of effector/memory T-cells and drive Th1 memory responses during mycobacterial infection.

### 3.7. T-Cells Activated by Rv3841-Stimulated DCs Inhibit Intracellular Mycobacterial Growth

**Figure 7:**
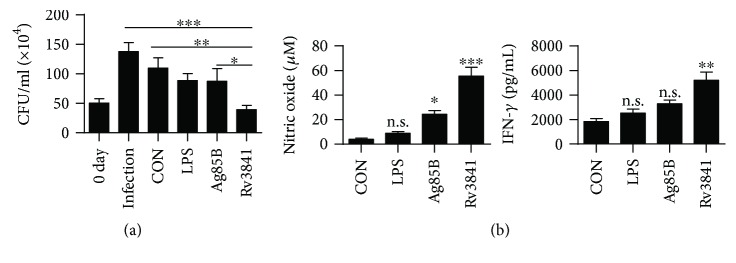
T-cells activated by Rv3841-treated DCs inhibit intracellular Mtb growth. Splenic T-cells or T-cells from Mtb-infected mice activated by unstimulated DCs, LPS-stimulated DCs, Ag85B-stimulated DCs, or Rv3841-stimulated DCs at a DC : T-cell ratio of 1 : 10 for 3 days were cocultured with BMDMs infected with Mtb. (a) Intracellular Mtb growth in BMDMs was determined at time point 0 (day 0) and 3 days after culturing with T-cells or without T-cells (control). (b) The NO and IFN-*γ* levels in culture supernatants were determined. The data are shown as mean ± SD (*n* = 3); ^∗^ *p* < 0.05, ^∗∗^ *p* < 0.01, or ^∗∗∗^ *p* < 0.001: a significant difference of BMDMs cocultured with T-cells from control BMDMs. n.s.: no significant difference.

On the basis of the above results, to confirm the involvement of Rv3841-stimulated DCs in the control of intracellular mycobacterial growth, we examined that T-cells activated by Rv3841-stimulated DCs could inhibit the bacterial growth within macrophages. Splenic CD4^+^ T-cells from Mtb-infected mice were activated by Rv3841-stimulated DCs for 3 d and then added to BMDMs. The plain addition of unactivated T-cells caused appreciable inhibition of intracellular mycobacterial growth. Of note, T-cells activated by Rv3841-stimulated DCs significantly inhibited the mycobacterial growth in BMDMs as compared to T-cells activated by control or LPS-stimulated DCs (Figure 7(a)). The importance of IFN-*γ* and nitric oxide (NO) in the control of mycobacterial growth is well established [38, 39]. Furthermore, IFN-*γ* and NO production, which are involved to antimycobacterial activity, were significantly elevated after the addition of T-cells activated by Rv3841-stimulated DCs in comparison with T-cells activated by control or LPS-stimulated DCs (Figure 7(b)). These results suggested that Rv3841-stimulated DCs can control intracellular mycobacterial growth via T-cell activation.

## 4. Discussion

The most important strategy for the development of a TB subunit vaccine is to identify the reliable antigens that can be included in the antigen combination. We have been reporting that the proteins activating DCs or macrophages are promising candidates for the development of an effective TB subunit vaccine [28, 29, 40]. In the present study, we demonstrate that the Rv3841 protein induces maturation of DCs and Th1 polarization of T-cells. PE_PGRS proteins induce maturation of DCs via the TLR2 pathway and stimulate CD4^+^ T-cell responses [16, 17]. Proteins Rv0315 and Rv0577 induce maturation and activation of DCs thus increasing the expression of proinflammatory cytokines and surface molecules involved in antigen presentation, leading to a Th1 immune response [41, 42]. In addition, a recent study showed that PE27 induces Th1-polarized immune responses of memory T-cells through functional activation of DCs [43]. Our previous study also showed that Rv2299c-maturated DCs promote Th1 immuneresponse with bactericidal activity and that a Rv2299c-fused protein has a vaccination potential [28]. Therefore, mycobacterial antigens inducing activation of DCs may lead to enhanced protective immunity against Mtb. For these reasons, using multidimensional fractionation of Mtb culture filtrate proteins, we have identified the proteins that have effects on DCs or macrophages [28, 29, 40]. Rv3841 was one of the active mycobacterial proteins identified during these experiments.

Mtb Rv3841 is a ferritin B participating in iron storage. The iron acquisition and iron storage pathways in Mtb perform crucial functions in the growth, virulence, and latency [25–27]. Rv3841 and Rv1876 (BfrA) exclusively work in iron homeostasis and storage and are upregulated under iron-rich and downregulated under iron-deprived conditions [44]. Accordingly, Rv3841 has an important role in iron storage and detoxification processes [25, 26]. A recent paper indicates that overexpression of the Rv3841 protein may be important for the survival and pathogenesis of aminoglycoside-resistant Mtb strains by modulating the effects of amikacin and kanamycin [45]. These observations suggest that Rv3841 is related to drug-resistant TB, which can be prevented just as XDR-TB can. Although Rv3841 is considered a causative antigen of TB pathogenesis, little is known about the cellular immune responses triggered by the Rv3841 protein. Our data show that a recombinant Rv3841 protein functionally induced maturation of DCs by augmenting the expression of cell surface markers and production of proinflammatory cytokines, which are downstream effects of the TLR4-related signaling pathways including MAPK and NF-*κ*B signal transduction. Furthermore, Rv3841-treated DCs (i) activated naïve T-cells, (ii) effectively polarized CD4^+^ and CD8^+^ T-cells so that they secrete IFN-*γ* and IL-2, and (iii) induced T-cell proliferation (Figure 4). Rv3841-maturated DCs specifically expanded a population of CD44^high^CD62L^low^CD4^+^ effector/memory cells among splenic T-cells collected from Mtb-infected mice, indicating that Rv3841 acts as a recall antigen in a Th1 memory response (Figure 6). Although a number of antigens from Mtb are known for their interaction with host cells, to the best of our knowledge, this is the first report showing that functions and signaling mechanisms of action of Rv3841 in DCs activate T-cell immunity. IFN-*γ* mediates antimicrobial action by activating phagocytes [46]—so that they rapidly ingest and degrade pathogenic microbes—and by activating inducible nitric oxide synthase (iNOS), which promotes microbicidal NO production [38, 47]. Our study indicates that the Rv3841-stimulated DCs can induce IFN-*γ* production in T-cells. T-cells activated by Rv3841-stimulated DCs enhanced NO production in infected macrophages. NO production is a part of the host defense against Mtb, particularly in the murine immune system [48]. These results suggest that Rv3841-stimulated DCs are important for a protective immune response against Mtb.

Collectively, our data reveal that Rv3841 enhances the immunostimulatory capacity of DCs to promote a Th1-polarized T-cell response in a TLR4-dependent pathway. Even if we did not supply direct evidence for vaccine effect of the recombinant Rv3841 protein, Rv3841 may be an excellent target for the rational design of effective TB vaccines.

This study offers novel data indicating that Rv3841 can drive Th1-polarized immune responses through DC maturation. Furthermore, Rv3841 induced a Th1-polarized memory CD4^+^ T-cell response during Mtb infection, suggesting that Rv3841 has a possibility as a successful vaccine candidate against TB.

## Supplementary Materials

Supplementary 1 Figure S1: recombinant Rv3841 induces DC maturation. (a) The purified recombinant Rv3841 protein was analyzed by SDS-PAGE with Coomassie blue staining (a) and Western blot analysis using an anti-His tag antibody (b). DCs were activated by the indicated concentration of Rv3841 or LPS (100 ng/mL) for 24 h. Bone marrow-derived dendritic cells (BMDCs) treated with the indicated concentration of Rv3841 for 24 h were analyzed by flow cytometry involving staining with an anti-CD11c antibody, annexin V, and PI. Staurosporine treatment served as a positive control. The results are representative of three experiments.

Supplementary 2 Figure S2: confirmation of endotoxin decontamination of the purified Rv3841. (A) The amount of residual LPS in the Rv3841 preparation was estimated using the Limulus amoebocyte lysate (LAL) test according to the manufacturer's instructions. (B) DCs were stimulated with Rv3841 denatured by boiling for 1 h at 100°C or digested with proteinase K (PK, 10 *μ*g/mL) for 1 h at 37°C. Alternatively, DCs were pretreated with polymyxin B (50 *μ*g/mL) for 1 h prior to stimulation of the DCs. LPS treatment (100 ng/mL) served as a control. After 24 h, the quantities of TNF-*α*, IL-1*β*, and IL-6 in the culture supernatant were measured by ELISAs. All the data are expressed as mean ± SD (*n* = 3), and statistical significance (^∗∗∗^ *p* < 0.001) is indicated for treatments compared to the controls, whereas treatments that showed no significant effect are indicated as “*n.s*.”

Supplementary 3 Figure S3: Rv3841-treated DCs stimulate T-cells to differentiate into the Th1 but not Th2 or Treg lineage. Transgenic OVA-specific CD4^+^ T-cells were isolated using MACS from OT-II mouse splenocytes and cocultured for 72 h with DCs pretreated with Rv3841 (10 *μ*g/mL) or LPS (100 ng/mL) and then pulsed with OVA_323–339_ (1 *μ*g/mL) to produce OVA-specific CD4^+^ T-cells. T-cells alone and T-cells cocultured with untreated DCs served as controls. Cytokine production was then assessed using intracellular FACS staining. The mean values ± SD (*n* = 3) are shown; ^∗^ *p* < 0.05, ^∗∗^ *p* < 0.01, or ^∗∗∗^ *p* < 0.001: a significant difference of treatment groups from the appropriate controls (T-cells + OVA_323–339_-pulsed DCs), as determined by one-way ANOVA. Treatments without a significant effect are indicated by “*n.s*.”

Supplementary 4 Figure S4: Rv3841-treated DCs induce expansion of the effector/memory T-cell population from lymph nodes of Mtb-infected mice. DCs from WT mice were treated with Rv3841 (10 *μ*g/mL) or LPS (100 ng/mL) and then cocultured for 3 days with T-cells from lymph nodes in Mtb-infected mice at the DC to T-cell ratio of 1 : 10. T-cells were stained with anti-CD4, anti-CD62L, and anti-CD44 monoclonal antibodies. A histogram is shown for gating of the labeled T-cells. Bar graphs show CD62L^low^CD44^high^ T-cells or CD62L^high^CD44^low^ T-cell populations. The mean values ± SD (*n* = 3) are shown; ^∗^ *p* < 0.05, ^∗∗^ *p* < 0.01, or ^∗∗∗^ *p* < 0.001: a significant difference of treatment groups from the appropriate controls, as determined by one-way ANOVA test. Treatments without a significant effect are indicated by “*n.s.*”
