# Supplementary figures and images for: Mycobacterium tuberculosis Protein Rv3841 Activates Dendritic Cells and Contributes to a T Helper 1 Immune Response
Source: J Immunol Res. 2018 Mar 15;2018:3525302. doi: 10.1155/2018/3525302 (PMC5875036; doi:10.1155/2018/3525302)

## Slide 1
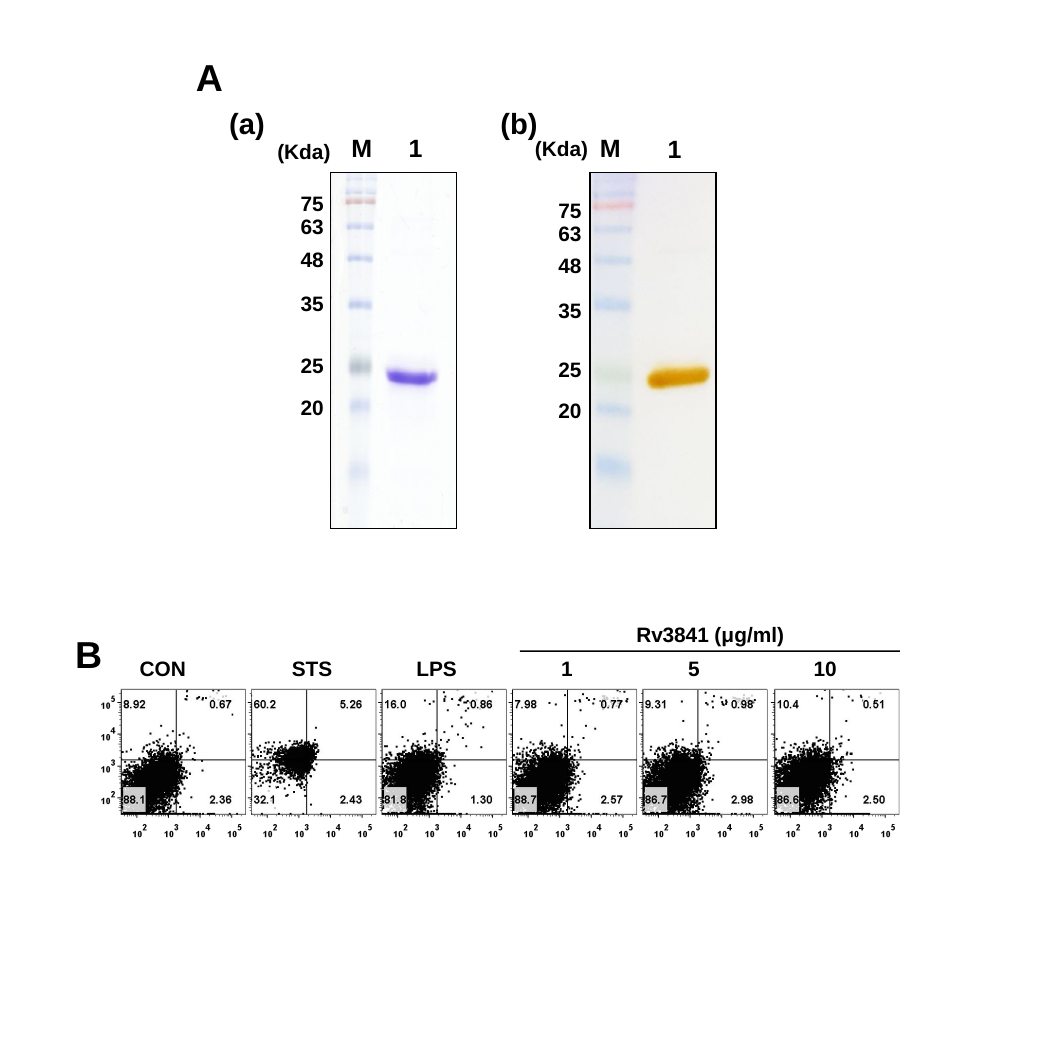

A
(a)
(b)
M
M
1
1
(Kda)
(Kda)
75
75
63
63
48
48
35
35
25
25
20
20
Rv3841 (μg/ml)
B
CON
STS
LPS
1
5
10

Supplement: Supplementary 1 — Figure S1: recombinant Rv3841 induces DC maturation. (a) The purified recombinant Rv3841 protein was analyzed by SDS-PAGE with Coomassie blue staining (a) and Western blot analysis using an anti-His tag antibody (b). DCs were activated by the indicated concentration of Rv3841 or LPS (100 ng/mL) for 24 h. Bone marrow-derived dendritic cells (BMDCs) treated with the indicated concentration of Rv3841 for 24 h were analyzed by flow cytometry involving staining with an anti-CD11c antibody, annexin V, and PI. Staurosporine treatment served as a positive control. The results are representative of three experiments. [file 3525302.f1.ppt]

## Slide 1
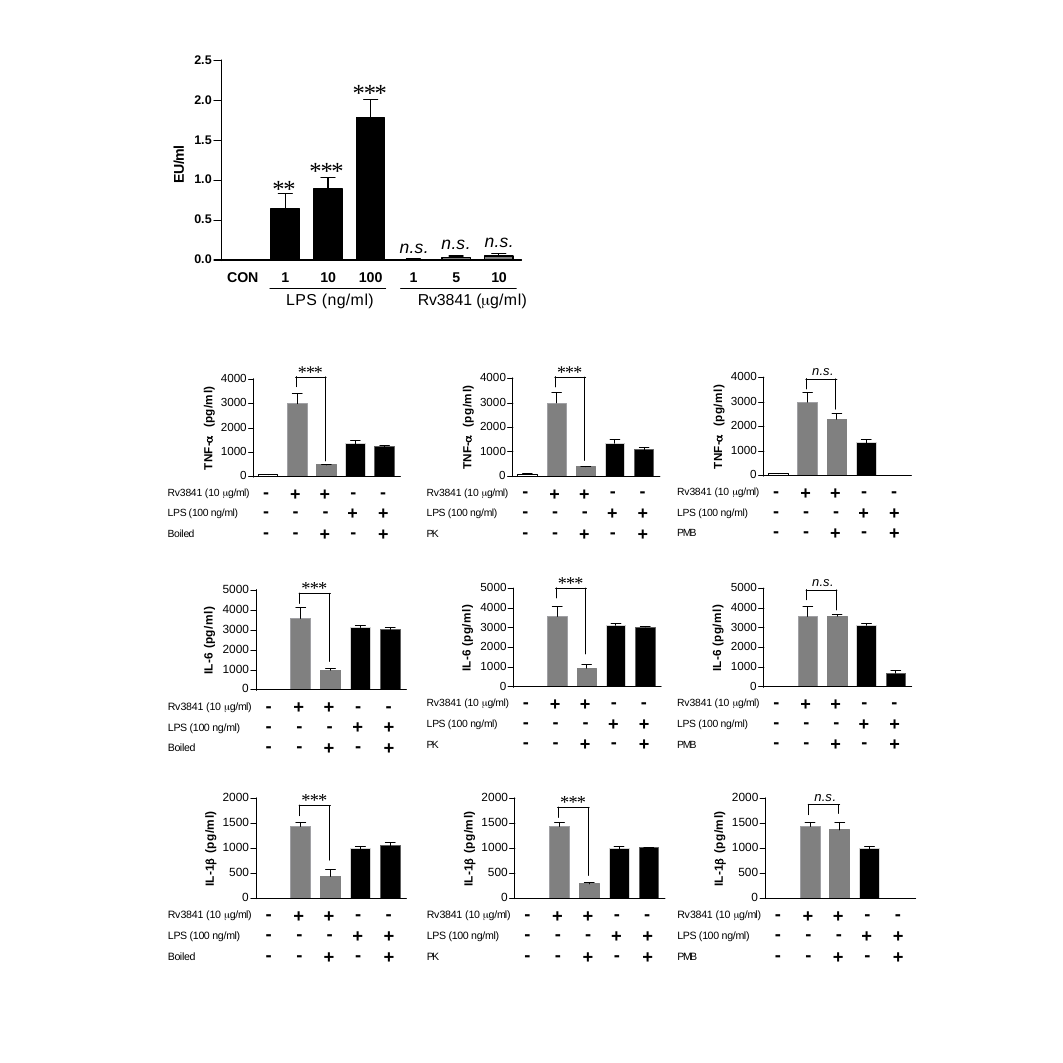

Supplement: Supplementary 2 — Figure S2: confirmation of endotoxin decontamination of the purified Rv3841. (A) The amount of residual LPS in the Rv3841 preparation was estimated using the Limulus amoebocyte lysate (LAL) test according to the manufacturer's instructions. (B) DCs were stimulated with Rv3841 denatured by boiling for 1 h at 100°C or digested with proteinase K (PK, 10 μg/mL) for 1 h at 37°C. Alternatively, DCs were pretreated with polymyxin B (50 μg/mL) for 1 h prior to stimulation of the DCs. LPS treatment (100 ng/mL) served as a control. After 24 h, the quantities of TNF-α, IL-1β, and IL-6 in the culture supernatant were measured by ELISAs. All the data are expressed as mean ± SD (n = 3), and statistical significance (∗∗∗ p < 0.001) is indicated for treatments compared to the controls, whereas treatments that showed no significant effect are indicated as “n.s.” [file 3525302.f2.ppt]

## Slide 1
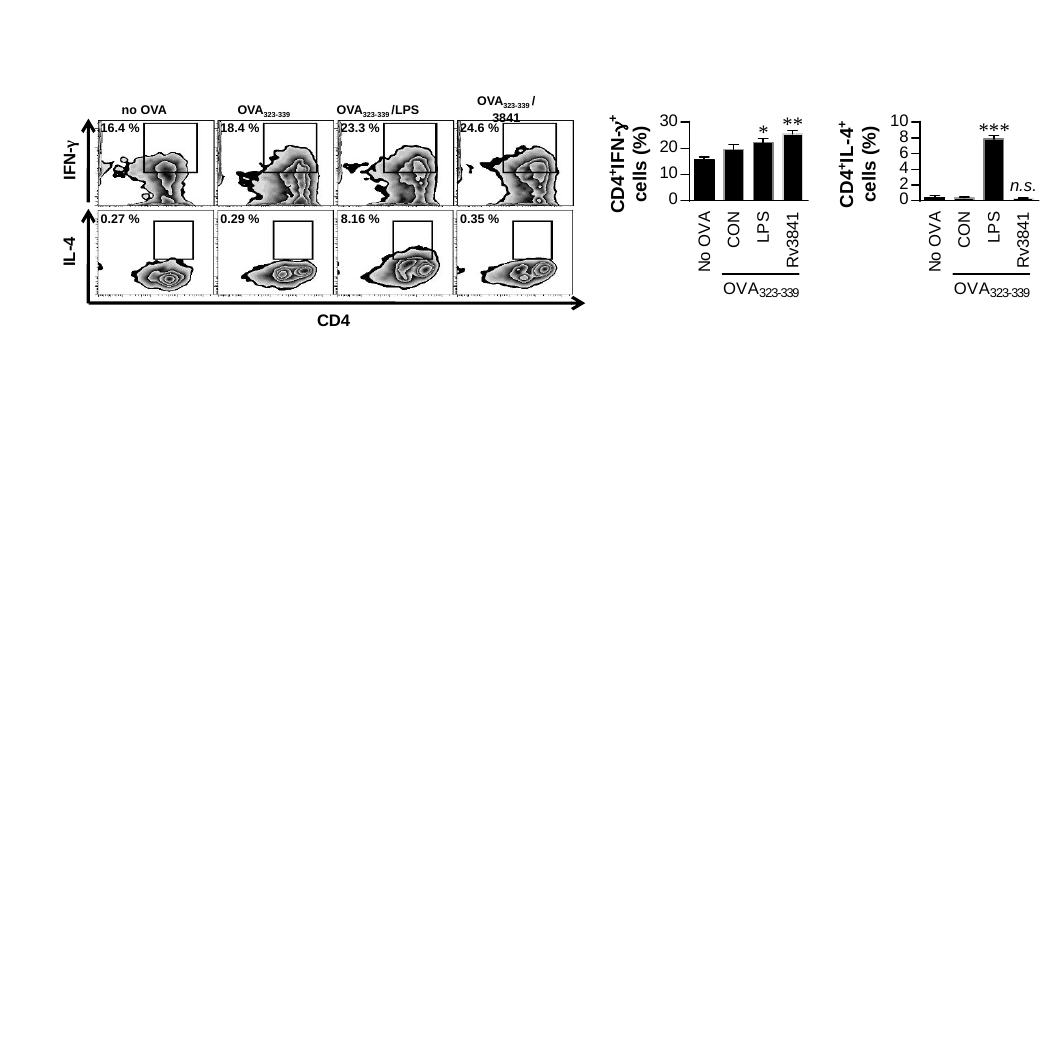

OVA323-339 /
3841
no OVA
OVA323-339
OVA323-339 /LPS
16.4 %
18.4 %
23.3 %
24.6 %
IFN-γ
0.27 %
0.29 %
8.16 %
0.35 %
IL-4
CD4

Supplement: Supplementary 3 — Figure S3: Rv3841-treated DCs stimulate T-cells to differentiate into the Th1 but not Th2 or Treg lineage. Transgenic OVA-specific CD4+ T-cells were isolated using MACS from OT-II mouse splenocytes and cocultured for 72 h with DCs pretreated with Rv3841 (10 μg/mL) or LPS (100 ng/mL) and then pulsed with OVA323–339 (1 μg/mL) to produce OVA-specific CD4+ T-cells. T-cells alone and T-cells cocultured with untreated DCs served as controls. Cytokine production was then assessed using intracellular FACS staining. The mean values ± SD (n = 3) are shown; ∗ p < 0.05, ∗∗ p < 0.01, or ∗∗∗ p < 0.001: a significant difference of treatment groups from the appropriate controls (T-cells + OVA323–339-pulsed DCs), as determined by one-way ANOVA. Treatments without a significant effect are indicated by “n.s.” [file 3525302.f3.ppt]

## Slide 1
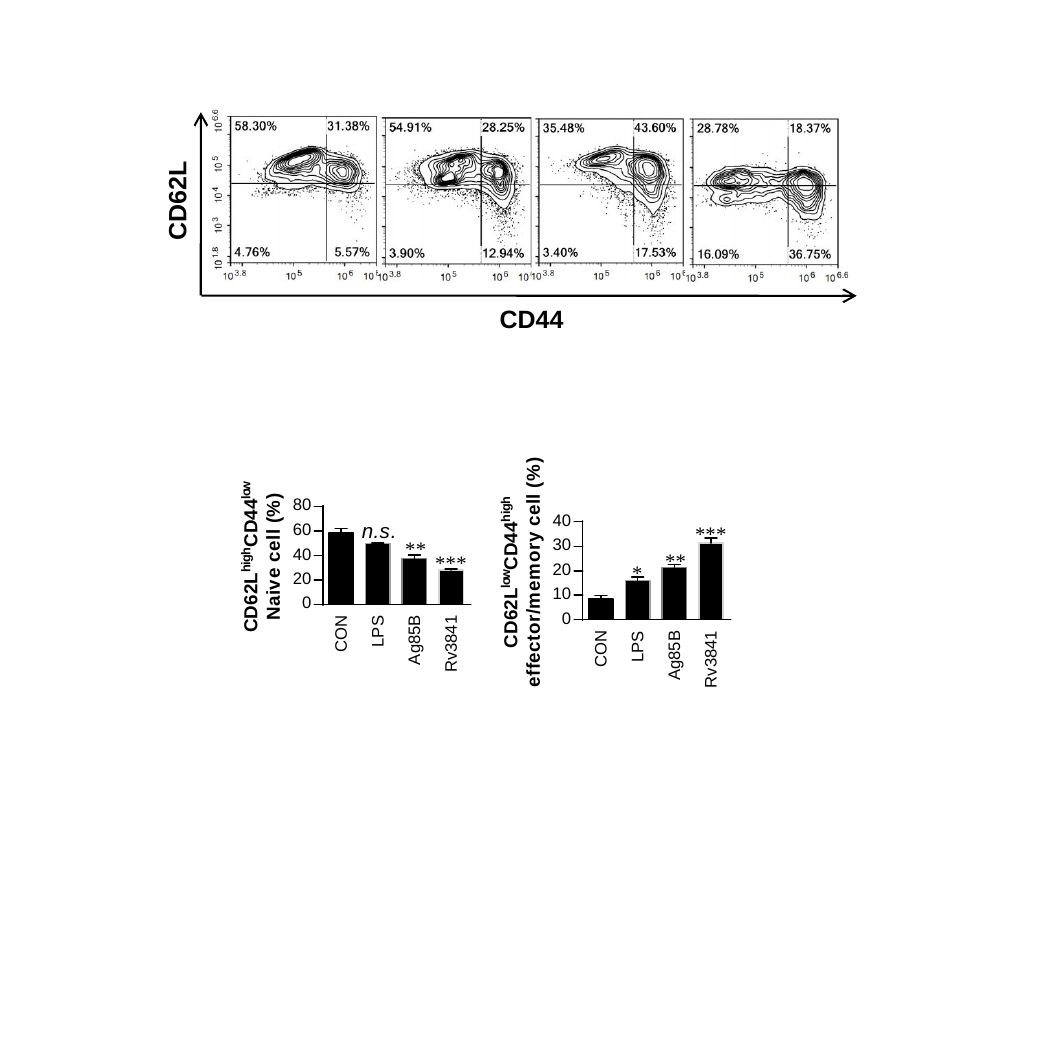

CD62L
CD44

Supplement: Supplementary 4 — Figure S4: Rv3841-treated DCs induce expansion of the effector/memory T-cell population from lymph nodes of Mtb-infected mice. DCs from WT mice were treated with Rv3841 (10 μg/mL) or LPS (100 ng/mL) and then cocultured for 3 days with T-cells from lymph nodes in Mtb-infected mice at the DC to T-cell ratio of 1 : 10. T-cells were stained with anti-CD4, anti-CD62L, and anti-CD44 monoclonal antibodies. A histogram is shown for gating of the labeled T-cells. Bar graphs show CD62LlowCD44high T-cells or CD62LhighCD44low T-cell populations. The mean values ± SD (n = 3) are shown; ∗ p < 0.05, ∗∗ p < 0.01, or ∗∗∗ p < 0.001: a significant difference of treatment groups from the appropriate controls, as determined by one-way ANOVA test. Treatments without a significant effect are indicated by “n.s.” [file 3525302.f4.ppt]
